# Monocytes and neutrophils expressing myeloperoxidase occur in fibrous caps and thrombi in unstable coronary plaques

**DOI:** 10.1186/1471-2261-9-27

**Published:** 2009-06-23

**Authors:** Fabio R Tavora, Mary Ripple, Ling Li, Allen P Burke

**Affiliations:** 1 Armed Forces Institute of Pathology, Washington, DC, 20306, USA; 2 University of Maryland Medical Systems, Baltimore, MD, 21201, USA

## Abstract

**Background:**

Myeloperoxidase (MPO) -containing macrophages and neutrophils have been described at sites of plaque rupture. The presence of these cells in precursor lesions to acute rupture (thin cap atheroma, or vulnerable plaque) and within thrombi adjacent to ruptures has not been described, nor an association with iron-containing macrophages within unstable plaques.

**Methods:**

We studied 61 acute ruptures, 15 organizing ruptures, 31 thin cap fibroatheromas, and 28 fibroatheromas from 72 sudden coronary death victims by immunohistochemical and histochemical techniques. Inflammatory cells were typed with anti-CD68 (macrophages), anti-BP-30 (neutrophil bactericidal glycoprotein), and anti-MPO. Iron was localized by Mallory's Prussian blue stain. In selected plaques alpha smooth muscle actin (DAKO, Carpinteria, CA, clone M0851) was performed.

**Results:**

MPO positive cells were present in 79% of ruptured caps, 28% of thin cap fibroatheroma, and no fibroatheromas; neutrophils were present in 72% of ruptures, 8% of thin cap fibroatheromas, and no fibroatheromas. Iron containing foam cells were present in the caps of 93% of acute ruptures, of 85% of organizing ruptures, 20% of thin cap atheromas, and 10% of fibroatheromas. MPO positive cells were more frequent in occlusive than non-occlusive thrombi adjacent to ruptures (p = .006) and were more numerous in diabetics compared to non-diabetics (p = .002)

**Conclusion:**

Unstable fibrous caps are more likely to contain MPO-positive cells, neutrophils, and iron-containing macrophages than fibrous caps of stable fibroatheromas. MPO-positive cells in thrombi adjacent to disrupted plaques are associated with occlusive thrombi and are more numerous in diabetic patients.

## Background

Plaque rupture is a major cause of coronary thrombosis, and is morphologically characterized by an interruption in a thin fibrous cap overlying a lipid rich core. [1-4] Often there are numerous macrophages infiltrating the fibrous cap of rupture plaques, suggesting a critical role in inciting plaque rupture. More recently, myeloperoxidase (MPO) expressing monocytes and neutrophils have been described at the site of plaque disruption, suggesting a role of MPO in plaque rupture. [5,6] However, the precise factor precipitating plaque rupture, as well as the morphologic and cellular differences that identify a vulnerable plaque (thin cap fibroatheroma) prone to rupture are currently not known.

Circulating monocytes produce hypochlorous acid by activation of MPO, increasing oxidative stress. [7,8] Recent evidence suggests that MPO-producing macrophages may persist in the atherosclerotic plaque and contribute to plaque instability. [9] In addition, serum levels of MPO seem to correlate with adverse risks in acute coronary syndrome patients.[10]

The purpose of this study was threefold: (1) to corroborate Sugiyama et al's and Naruko's finding that MPO positive cells including neutrophils are increased at the site of plaque instability; [5,6] (2) to quantitate MPO positive cells and neutrophils for the first time in thin-cap fibroatheroma; and (3) to correlate numbers of MPO positive cells in thrombi adjacent to ruptures with thrombus characteristics and risk factors.

## Methods

Autopsy sections of coronary artery plaques were selected from patients dying with severe coronary atherosclerosis. Inclusion for study included apparent natural cardiac deaths at the time of autopsy, with drug-related deaths subsequently excluded in cases of positive toxicology. Cases were seen in consultation at the Armed Forces Institute of Pathology from the Office of the Chief Medical Examiner in Baltimore, Maryland, under institutional review board approval. Hearts with severe coronary artery disease (≥ 75% cross section luminal narrowing of ≥ 1 epicardial artery) were included for study. Sections selected for study included 135 sections from 72 patients, namely 28 fibroatheromas, 31 thin-cap fibroatheromas, 61 acute plaque ruptures, 15 healing plaque ruptures. Plaque types were defined as previously described [11-16].

The distribution of lesions per arterial segment is presented on Table 1. Healing rupture denoted areas of acute thrombus overlying plaque disruption, with endothelial cell organization of portion of the fibrin thrombus with persistence of fibrin within the thrombus. [12]. Risk factors were determined by clinical history obtained from investigators and by serum corroboration when necessary. No cases of intra-coronary intervention were included in the study.

**Table 1:** Distribution of lesions by arterial segment

Arterial segment	Acute ruptures (n = 61)	Healing ruptures (n = 15)	Thin cap fibroatheroma (n = 31)	Stable plaques (n = 27)
Left main	2	0	0	0
Proximal LAD	16	5	6	10
Mid LAD	8	0	2	2
Distal LAD	0	0	1	1
Left diagonal	0	0	1	0
Left circumflex	13	2	3	4
Obtuse marginal	1	1	1	1
Proximal RC	8	2	8	6
Mid RC	7	5	5	2
Distal RC	5	0	2	1
PDA	1	0	2	0

LAD = left anterior descending; LOM = left obtuse marginal; RC right coronary; PDA = posterior descending

Immunohistochemical inflammatory markers studied were CD68 (KP1 clone, Dako, Carpinteria, CA, dilution 1:50, pan-macrophage marker), BP 30/Cathepsin G (R & D Systems, Minneapolis, 1:80, neutrophil marker), and MPO (Biodesign, Kennebunk ME, dilution 1:250). Iron stain was performed using standard Perls modification of Mallory Prussian blue iron histochemical stain. In selected plaques alpha smooth muscle actin (DAKO, Carpinteria, CA, clone M0851) was performed at 1:400 dilution. Quantitation of cellular elements was performed in the fibrous cap region by computerized morphometric measurements (IPLab SpectrumTM image processing software, Signal Analytics Corporation, Vienna, VA). Comparisons of two means was performed using Student's T test, and of multiple categories was performed using ANOVA means table with Fisher's post hoc testing. Non-parametric comparison was performed using Mann-Whitney comparison means testing. Statistical analysis was performed using SAS software (Cary, NC).

## Results

**Figure 1:**
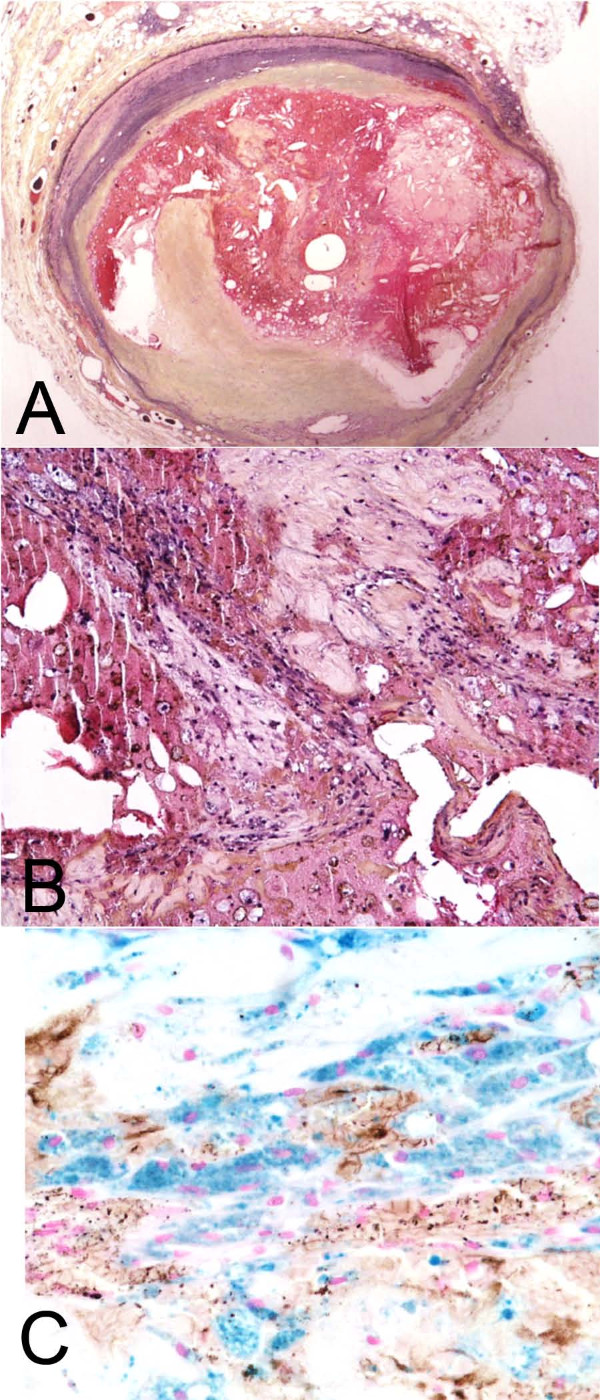
**Acute plaque rupture with iron-containing macrophages at the rupture site**. A. Low magnification of plaque rupture with occlusive thrombus. Movat pentachrome. B. Higher magnification of A showing rupture site demonstrating fragmented cap (top) and thrombus below. C. Perl's iron stain of the same area as B, demonstrating a large number of hemosiderin-laden macrophages at the rupture site.

**Figure 2:**
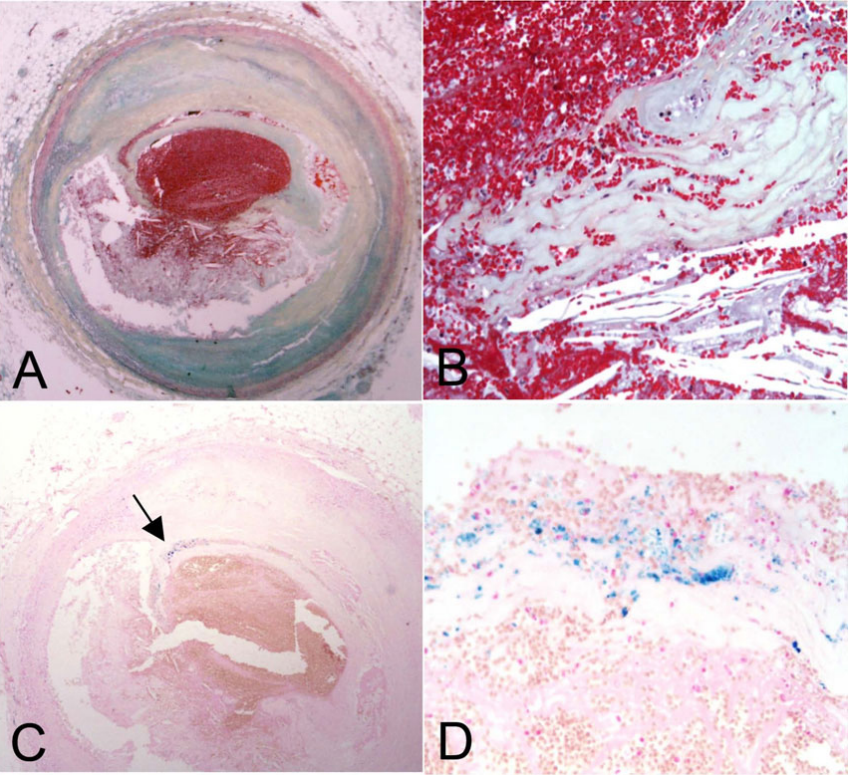
**Acute plaque rupture with iron-containing macrophages at the rupture site**. A. Low magnification of plaque rupture with occlusive thrombus. Movat pentachrome. B. Higher magnification of A showing rupture site with prominent cholesterol crystals. C. Perl's iron stain, low magnification demonstrating hemosiderin-laden macrophages at the rupture site (arrow), seen at higher magnification in D.

The patient age ranged from 37 to 82 (mean, 52 years), and there were 66 male and 6 females patients. Seventy-three percent were white and 26% black. The heart weights ranged from 204 to 875 (mean 511 g). Risk factors included 10 diabetic patients, 22 hypertensive, 37 hypercholesterolemic and 36 smokers. There were no statistical differences between the three groups (acute rupture, healing rupture, stable plaque) in regards to sex, age, gender, or heart weight (Table 2). Risk factors and demographic data are presented in Table 2, as well as distribution of sampled plaque types by culprit plaque morphology. Of the 72 patients, culprit plaques were 51 acute ruptures (Figures 1 and 2), 5 healing ruptures, and 16 stable plaques without plaque disruption. The plaques sampled were 61 ruptures, 15 healing ruptures, 31 thin cap fibroatheromas, and 28 fibroatheromas with thick caps.

**Table 2:** Patient demographics, 135 plaques from 72 sudden coronary deaths

	Culprit plaque			Totals
		
	Acute rupture	Healing rupture	Stable plaque	
N	51	5	16	72
Age, years ± SD	52 ± 11	52 ± 10	48 ± 8	51 ± 11
Men: women	47:4	5:0	14:2	66:6
White:Black	38:13	3:2	12:4	53:19
Heart weight, g ± SD	504 ± 127	605 ± 125	466 ± 87	511 ± 134
Diabetic	9	0	1	10
Hypertensive	13	4	5	22
Hypercholesterolemic	25	3	9	37
Smokers	26	3	7	36
Number of plaques studied	100*	7	28	135
Acute ruptures	61	0	0	61
Healing ruptures	8	0	0	15
Thin cap FA	20	7	11	31
FA	11	0	17	28

FA = fibroatheroma

* in 10 hearts, 2 rupture sites > 3 mm separated were sampled

**Figure 3:**
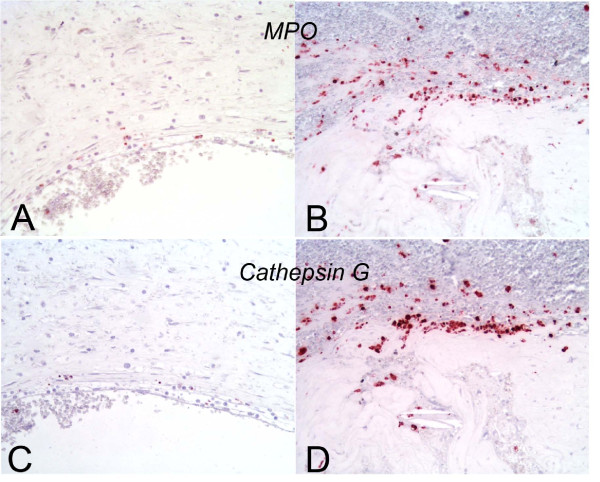
**MPO positive cells and neutrophils proximal to and at acute rupture sites**. A. 3 mm proximal to an acute rupture site there were only scattered MPO-positive macrophages at the luminal surface. B. The rupture site demonstrates numerous MPO positive cells. C and D are corresponding images of the sites of A and B, respectively, demonstrating that many of the MPO positive cells express cathepsin G, a neutrophil marker.

**Figure 4:**
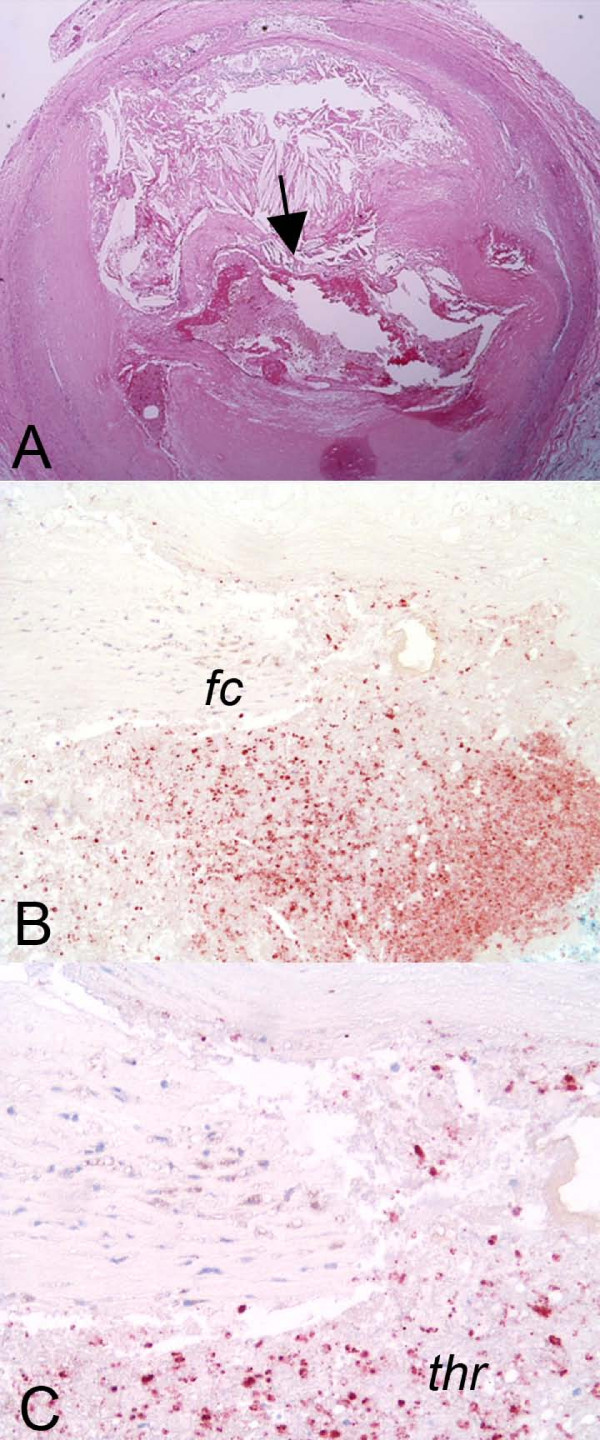
**MPO positive cells in the rupture site thrombus**. A demonstrates a low magnification of rupture of a large necrotic core. The arrow is the rupture site shown at higher magnification at B, which shows the fibrous cap (fc) above a thrombus rich in MPO-positive cells (immunohistochemical stain for myeloperoxidase). C is a slightly higher magnification demonstrating the thrombus (thr) just adjacent to the torn fibrous cap.

Table 3 demonstrates the density of macrophages (identified by CD 68 immunostaining), neutrophils (identified by cathepsin G immunostaining), MPO containing macrophages/neutrophils (Figures 3 and 4), and iron-containing macrophages within the fibrous caps of fibroatheromas, thin cap fibroatheromas, and acute and healing ruptures.

**Table 3:** Inflammatory infiltrates, fibrous cap

Cap type	Macro-phages/mm^2^	% with any neutro-phils	Neutro-phils/mm^2^	% with any MPO + cells	MPO + cells/mm^2^	% with any iron + macro-phages	Iron + macro-phages/mm^2^
Thick fibrous cap (fibro-atheroma, n = 28)	149 ± 199	0	0	0	0	10	1.2
Thin cap (n = 31)	399 ± 238	8	2 ± 2	28	13 ± 5	32	20 ± 13
Rupture (n = 61)	416 ± 287	79	72 ± 22	79	117 ± 22	93	88 ± 44
Healing rupture (n = 15)	304 ± 269	35	5 ± 5	60	47 ± 20	85	90 ± 41
P value*	P < .0001	P < .0001	P = .001	P < .0001	P < .0001	P < .0001	P < .0001

* ANOVA means table with Fisher's post-hoc testing

There was significant increase in macrophages in thin cap fibroatheromas, ruptures, and healing ruptures vs. fibroatheromas (p < .0001). No fibroatheroma had intracap neutrophils, vs. 8% of thin cap fibroatheromas, 79% of acute ruptures, and 35% of healing ruptures (p < .0001). There was no difference in intracap MPO positive macrophages or neutrophils between thin cap fibroatheromas with culprit plaque acute ruptures as compared to stable plaque. MPO cells were absent in fibroatheromas, but present in 28% of thin cap fibroatheromas, 79% of ruptures, and 60% of healing ruptures (p < .0001). Iron containing macrophages were present similarly more frequently in acute and healing ruptures than fibroatheromas and thin cap fibroatheromas (p < .0001).

Occlusive thrombi were longer than non-occlusive (Table 4), although there was no difference in length of necrotic core. Density of cap MPO macrophages/neutrophils did not differ between occlusive and non-occlusive thrombi; however, adjacent thrombus had a higher concentration of MPO and CD68 macrophages than non-occlusive thrombi (p < .01, Table 4). Density of intracap macrophages overlying disrupted plaques (acute or organizing ruptures) did not vary by risk factor; however, the density of MPO positive cells was greater in diabetics with borderline significance for smokers (Table 5). Acute ruptures characterized by thin caps without actin positive cells, variable numbers of actin positive smooth muscle cells at the base of the plaque towards the internal elastic laminae (IEL). Healing ruptures, the rupture site characterized by fibrin, granulation tissue and none to few actin positive cells, again variable numbers of actin positive smooth muscle cells at the base of the plaque towards the IEL. In thin cap fibroatheromas, the cap itself contained no smooth muscle cells, but there were variable numbers of actin positive smooth muscle cells at the base of the plaque towards the IEL."

**Table 4:** Plaque ruptures: Inflammatory cell density, luminal thrombi, and correlation with necrotic core characteristics

	Length, mm	Core length, mm	Thrombus MPO + cell density/mm^2^	Thrombus CD68 macro-phage density/mm^2^	Cap MPO + cell density/mm^2^	Cap CD68 macro-phage density/mm^2^
Occlusive	8.3 ± 4.2	8.9 ± 5.1	366 ± 80	424 ± 119	164 ± 85	352 ± 132
Non-occlusive	2.9 ± 2.4	9.3 ± 6.6	45 ± 32	83 ± 72	26 ± 11	540 ± 173
P value	.01	0.9	.006	.004	.2	.4

**Table 5:** Acute and organizing ruptures: affect of risk factors on density of MPO + cells in cap and thrombus

Risk factor	Cap CD68 + macrophages/mm^2^	Thrombus CD68+ macrophages/mm^2^	Cap MPO + cells/mm^2^	Thrombus MPO + cells/mm^2^
Diabetic	386 ± 64	239 ± 88	91 ± 31	510 ± 148
Non diabetic	445 ± 45	475 ± 245	110 ± 26	137 ± 36*
Smoker	394 ± 53	359 ± 111	87 ± 16	340 ± 96
Non-smoker	479 ± 56	216 ± 142	126 ± 40	119 ± 41**
Hypertensive	433 ± 72	333 ± 205	65 ± 13	330 ± 147
Non-hypertensive	429 ± 49	164 ± 61	106 ± 24	178 ± 65
History of dyslipidemia	412 ± 56	302 ± 141	78 ± 17	271 ± 90
No history of dyslipidemia	445 ± 60	132 ± 52	106 ± 30	134 ± 60

* p = .002

** p = .06

## Discussion

The current study corroborates work by others, showing increased MPO-containing cells and neutrophils in plaque rupture sites. [5,6] It reveals new information regarding MPO content comparing plaque subtype, especially small numbers in thin cap atheroma. MPO co-localizes with iron containing macrophages on the surface and its density in thrombotic lesions correlates well with thrombus size. The numbers of MPO containing cells are also significantly higher in patients with diabetes and smoking history, creating a link between risk factors and histomorphological findings.

MPO has emerged as a potential participant in the promotion of atherosclerosis. [17] It is stored and secreted from activated neutrophils and monocytes, and is an important component in degranulation material of leukocytes, critical in human innate host defenses. [5,6] The MPO role in atherosclerosis initiation and propagation is related to its potential to activate lipid peroxidation and promoting post-translational modification of target proteins.[18,19] MPO catalyzes LDL oxidation and releases HOCl, degrading extracellular matrix. Recent studies have found that MPO is capable of promoting oxidation of lipoproteins which could lead to the increase in cholesterol deposit and formation of foam cells in fatty streaks.[18,20]

Tissue localization of MPO has been described in a variety of inflammatory conditions,[19] MPO was found to be increased in human atheromas more than a decade ago.[21] More recently, studies have focused on the presence of MPO not only in early lesions, but also in acute complications of atherosclerosis and plaque vulnerability.[9] The link between MPO levels and CAD is strongly supported by epidemiological studies. Individuals who have high MPO levels are more likely to demonstrate abnormal coronary angiograms compared to controls.[22] Individuals with MPO deficiency have some protection against CAD and others harboring a polymorphism that decreases MPO expression have markedly reduced rates of CAD, myocardial infarction and cardiac death. [23-25]

The current study corroborates the findings of Naruko et al who analyzed 126 coronary sections using immunohistochemical studies for macrophages (CD66b), neutrophils (CD11b) and myeloperoxidase. [5] The population in their study was composed of material from autopsies and atherectomy procedures. In the control of patients dying form non-cardiac deaths, MPO was not indentified in any case, whereas ruptured or eroded plaques had MPO positivity mostly in neutrophils, and only occasionally in macrophages. A similar approach was used by Sugiyama et al, who found increased numbers of MPO-containing macrophages in eroded and ruptured plaques, with little to no MPO in fatty streaks. The expression of MPO was also variably dependent on the stage of atherosclerotic lesions, with special high expression with fibrous caps and activated plaques. [6] Our finding of higher MPO expression in patients with diabetes substantiates clinical studies that measured MPO in plasma of diabetic patients versus controls. [26,27]

It is known that free iron in the plasma catalyzes lipid peroxidation and this reaction has been involved in the development of atherosclerosis in the way of hydroxyl radical formation by neutrophils requiring exogenous iron.[28] Iron is stored as either ferritin or hemosiderin. Ferritin consists of an outer protein shell with iron complexed within the core. It has been postulated that either MPO or lactoferrin could inhibit the formation of the hydroxyl radical upon neutrophil degranulation. Since it can be seen by light microscopy as gold-brown granules and is demonstrated by the Prussian blue stain, we assessed positivity of iron containing cells in culprit lesions and found that these were more frequently present in acute and healing ruptures than fibroatheromas and thin cap fibroatheromas. Although the molecular relationship between iron and myeloperoxidase is unknown, MPO contains an iron porphyrin prosthetic group. In other inflammatory conditions, such as asthma, there seems to be an association between levels of MPO and iron, and it is suggested they may act in a concerted manner in the pathogenesis of the disease. [29] While our findings of elevated iron and MPO do not elucidate its possible mechanistic relationship, it brings new data to the discussion of the pathogenesis of atherosclerosis progression.

## Conclusion

MPO localizes in macrophages and neutrophils at the site of plaque rupture, as has been previously demonstrated, and only occasionally in precursor thin cap atheroma. A causative association between MPO and rupture has not been established but remains a possibility that requires further study. Localization of iron at rupture sites is a novel finding that may have implications for imaging of unstable plaques. Whether there is a link between iron deposits at rupture sites and MPO likewise remains an area for investigation.

## Competing interests

The authors declare that they have no competing interests.

## Authors' contributions

LL and MR collected all cases and participated on data analysis. LL and AB participated in the design of the study. FT and AB performed the statistical analysis. AB and FT conceived of the study, and participated in its design and coordination and helped to draft the manuscript. All authors read and approved the final manuscript.

## Pre-publication history

The pre-publication history for this paper can be accessed here:


